# Associations between food preferences, micronutrient insufficiency, and autonomic function in adolescents with a recent history of vasovagal syncope

**DOI:** 10.3389/fnut.2026.1783737

**Published:** 2026-04-09

**Authors:** Tetiana Kovalchuk, Oksana Boyarchuk

**Affiliations:** 1 Department of Pediatrics, Ivan Horbachevsky Ternopil National Medical University of the Ministry of Health of Ukraine, Ternopil, Ukraine; 2 Department of Pediatrics and Pediatric Surgery, Ivan Horbachevsky Ternopil National Medical University of the Ministry of Health of Ukraine, Ternopil, Ukraine

**Keywords:** 25-hydroxyvitamin D, cobalamin, folate, food preferences, heart rate variability, pediatric vasovagal syncope, pyridoxine

## Abstract

**Aim of the study:**

This study aimed to investigate the interrelationships between food preferences, micronutrient status, and autonomic function in adolescents with vasovagal syncope (VVS), exploring how dietary behaviour may contribute to syncope susceptibility.

**Materials and methods:**

We enrolled 138 adolescents with VVS and 78 age-matched healthy controls (13–17 years). Clinical assessment included syncope history, Modified Calgary Syncope Score (MCSS), and 24-h Holter monitoring for heart rate variability (HRV) analysis (SDANN, RMSSD, pNN50, TP, VLF, LF, HF, and LF/HF). Fasting serum levels of vitamins B6, B12, folate, and 25-hydroxyvitamin D (25(OH)D) were measured via ELISA. Food preferences were evaluated using a validated Ukrainian version of the Food Preference Questionnaire (FPQ) for adolescents and adults, covering vegetables, fruits, meat and fish, dairy, snacks, and starches. Data were analyzed using multiple linear regression, with assessment of FPQ reliability, construct, and discriminant validity.

**Results:**

Adolescents with VVS exhibited lower serum B6, B12, and 25(OH)D compared with controls, while folate levels were similar. HRV analysis demonstrated increased TP alongside elevated LF/HF ratios, suggesting altered autonomic regulation characterized by relative sympathetic predominance and reduced parasympathetic modulation at baseline. The FPQ demonstrated high reliability and validity. VVS participants reported lower preferences for vegetables, meat and fish, and starches (all FDR-corrected), with a trend toward lower fruit preference that did not survive correction. Regression analyses showed that higher vegetable preference was associated with increased folate and cobalamin, whereas meat and fish preference was positively associated with B6. Vegetable preference was negatively associated with HF, while meat and fish and dairy preferences were negatively associated with SDANN and RMSSD. Dairy preference showed a negative association with B6 and multiple HRV parameters, while snack and starch preference had modest or no associations. Food preferences were generally weakly related to syncope severity, with only starch preference showing a significant association with MCSS scores after FDR correction.

**Conclusion:**

Adolescents with VVS exhibit distinct food preference patterns linked to micronutrient insufficiencies and altered HRV, suggesting an interaction between diet, nutrient status, and autonomic regulation. Integrating nutritional assessment and targeted interventions into clinical management may improve autonomic balance and reduce syncope burden. Future longitudinal and interventional studies are needed to clarify causal pathways and evaluate the efficacy of dietary and supplementation strategies in mitigating VVS episodes.

## Introduction

Vasovagal syncope (VVS) is the most common cause of transient loss of consciousness in adolescents, typically triggered by prolonged standing, emotional stress, or postural changes. Although generally benign, recurrent episodes can substantially impair daily functioning and quality of life. The underlying mechanism involves autonomic dysregulation—an imbalance between sympathetic and parasympathetic activity—often reflected in altered heart rate variability (HRV) indices (1, 2).

Emerging evidence indicates that micronutrient deficiencies, particularly of vitamin D and B-group vitamins, may contribute to this autonomic imbalance and influence vascular reactivity. Low serum 25(OH)D levels have been linked with reduced parasympathetic modulation and disturbed circadian blood pressure rhythm in adolescents with VVS (2, 3). Similarly, deficiencies in vitamins B6, B12, and folate may impair neurotransmitter synthesis, endothelial function, and homocysteine metabolism, potentially increasing syncope susceptibility (4–6). Interventional data further suggest that combined vitamin B and D supplementation can improve HRV parameters and decrease the frequency of syncopal episodes (7).

In addition to biochemical factors, food preferences and eating behaviour appear to be associated with both micronutrient status and autonomic regulation (8, 9). Adolescents with VVS have been shown to exhibit lower enjoyment of food and greater satiety responsiveness, which correlate with decreased neurotropic vitamin levels and elevated homocysteine concentrations (10). Although food preferences do not directly measure dietary intake, they have been shown to be a significant predictor of habitual food choices and dietary patterns in children and adolescents, particularly when access and availability are not major barriers (11, 12). Therefore, food preferences may serve as a behavioral indicator reflecting underlying dietary tendencies associated with micronutrient status.

Despite increasing research interest, comprehensive studies simultaneously assessing food preferences, vitamin status, and HRV in adolescents with VVS remain scarce. Understanding these interrelationships could help identify modifiable behavioral and nutritional risk factors relevant for prevention and management. Therefore, this study aimed to characterize food preference patterns in adolescents with a recent history of VVS and to explore their associations with serum vitamin levels (vitamin D, B6, B12, folate) and HRV parameters reflecting autonomic balance. We hypothesized that adolescents with VVS would display distinct food preference profiles linked to micronutrient insufficiencies and altered HRV, suggesting an integrated mechanism contributing to syncope predisposition.

## Materials and methods

### Study population

The study enrolled 138 adolescents with vasovagal syncope (VVS) and 78 age-matched healthy controls (13–17 years). At enrollment, body weight was measured using a calibrated electronic scale and height using a wall-mounted stadiometer; body mass index (BMI) was calculated as weight (kg) divided by height squared (m^2^). Demographic covariates including sex (male/female), age (years), and area of residence (rural/urban) were recorded for all participants and included as independent variables in regression analyses, as these factors are known to influence food preferences in pediatric populations.

Inclusion criteria for VVS were syncope triggered by pain, fear, or prolonged standing with a typical prodrome, at least one episode in the preceding month, normal orthostatic testing, absence of structural heart disease or arrhythmogenic ECG features, no epileptiform EEG activity, and no other identifiable cause. Controls had no history of VVS and normal clinical and laboratory findings.

Exclusion criteria for all participants included overweight/obesity, hypertension, anemia, malabsorption, chronic kidney disease, acute illness or exacerbation of chronic conditions, vitamin B or D supplementation within the past year, enrollment between May and August, inability to read/write Ukrainian, or lack of informed consent. The study was approved by the Ethics Committee of Ivan Horbachevsky Ternopil National Medical University, and written informed consent was obtained from parents or legal guardians, and assent was obtained from all adolescent participants.

### Clinical assessment

The total number of syncopal events was obtained from patient and caregiver reports. For the most recent episode, presyncope, syncope, and postsyncope durations were recorded in minutes. Clinical features of VVS were further evaluated using the Modified Calgary Syncope Score (MCSS), applied consistently by trained investigators.

#### HRV analysis

All participants underwent 24-h Holter monitoring using a three-channel SDM3 recorder (Ukraine). ECG signals were recorded at a sampling rate of 1,000 Hz. Recordings were transferred to CardioSens software (Ukraine) for automated R-wave detection and NN interval extraction. All recordings were visually inspected by a trained investigator blinded to clinical data. Artifacts, ectopic beats (premature atrial and ventricular complexes), and segments with excessive noise were identified and corrected manually. Recordings with more than 5% ectopic beats or fewer than 22 h of analyzable data were excluded from analysis. Time-domain parameters included SDANN, RMSSD, and pNN50; frequency-domain parameters included TP, VLF (0.003–0.04 Hz), LF (0.04–0.15 Hz), HF (0.15–0.40 Hz), and LF/HF ratio. Frequency-domain analysis was performed using fast Fourier transform (FFT). HRV analysis was conducted in accordance with the guidelines of the Task Force of the European Society of Cardiology and the North American Society of Pacing and Electrophysiology (13).

#### Serum vitamin assessment

Fasting venous blood (5 mL) was collected, centrifuged, and serum stored at −80 °C. Concentrations of pyridoxine (B6), folate (B9), cobalamin (B12), and 25-hydroxyvitamin D (25(OH)D) were measured using ELISA.

#### Assessment of food preferences

Food preferences for 62 individual items were evaluated using the Food Preference Questionnaire (FPQ) for adolescents and adults (14). With permission from the original developers—Dr. Rebecca J. Beeke, Dr. Alison Fildes, and Dr. Andrea Smith—the FPQ was translated from English into Ukrainian following a rigorous linguistic validation process, comprising forward translation, back translation, cognitive interviews, and proofreading. This process produced a validated Ukrainian version of the FPQ, and all validation reports were submitted to co-developer Dr. A. Smith.

The FPQ was self-administered; adolescents completed the questionnaire independently in a quiet room under the supervision of a trained investigator, without parental assistance. Food preferences were rated on a 5-point Likert scale (1 = “dislike very much”; 2 = “dislike a little”; 3 = “neither like nor dislike”; 4 = “like a little”; 5 = “like a lot”), and categorized into six groups: vegetables (18 items), fruits (7 items), meat and fish (12 items), dairy (10 items), snacks (9 items), and starches (6 items).

### Statistical analysis

Data were analyzed using SPSS 26.0 for Windows. Continuous variables are presented as mean ± SD for normally distributed data and as median (IQR) for non-normal data, while categorical variables are reported as *n* (%). Between-group comparisons were performed using the Student’s *t*-test for normally distributed continuous variables, the Mann–Whitney *U* test for non-normally distributed continuous variables, and the *χ*^2^ test for categorical variables. Associations between food preferences and demographics, clinical syncope characteristics, serum vitamin levels, and HRV parameters were analyzed using multiple linear regression, with standardized regression coefficients (*β*), standard errors (SE), and *p*-values reported. Prior to interpretation, multiple linear regression models were evaluated for multicollinearity using variance inflation factors (VIF), and model assumptions were assessed by inspection of residual plots to confirm linearity, homoscedasticity, and normality of residuals. The Benjamini–Hochberg false discovery rate (FDR) correction was applied to adjust for multiple comparisons. A *p*-value < 0.05 was considered statistically significant.

The Food Preference Questionnaire (FPQ) for adolescents and adults was evaluated for data quality, including the percentage of missing responses per item and the presence of floor and ceiling effects across all scales. Internal consistency was assessed using Cronbach’s *α*, with values ≥0.70 considered acceptable. Discriminant validity was examined using Hedge’s g to quantify differences between VVS and control groups for each FPQ scale. Construct validity was confirmed via exploratory factor analysis, with factor loadings evaluated to verify the underlying structure of the questionnaire.

## Results

The study included 138 adolescents with VVS and 78 healthy controls, with no significant differences in age (15.1 ± 1.2 vs. 14.8 ± 1.1 years, *p* = 0.634), sex, residence, or BMI. Adolescents with VVS experienced a median of 2.0 [1.0; 4.0] syncopal episodes, with a mean MCSS score of −3.2 ± 1.2 points. HRV analysis revealed higher TP in the VVS group (7,723.0 [4,368.0; 11,748.0] vs. 5,471.0 [3,853.0; 7067.5] ms^2^, *p* = 0.012) and an elevated LF/HF ratio (1.3 [0.9; 2.1] vs. 1.1 [0.9; 1.7], *p* = 0.039). Biochemical analysis showed significantly lower serum pyridoxine (8.9 ± 2.7 vs. 14.6 ± 4.2 μg/L, *p* < 0.001), cobalamin (315.9 ± 93.5 vs. 428.5 ± 79.1 ng/L, *p* < 0.001), and 25(OH)D (19.6 ± 4.5 vs. 31.6 ± 6.2 ng/mL, *p* < 0.001) in the VVS group, with folate levels comparable between groups (4.2 ± 1.3 vs. 4.5 ± 1.4 μg/L, *p* = 0.252; Table 1).

**Table 1:** Demographic, clinical and biochemical characteristics of patients.

Characteristics	VVS group	Control group	*p*
Cases (*n*)	138	78	–
Males/Females (*n*)	72/66	36/42	0.396
Age, years (M ± SD)	15.1 ± 1.2	14.8 ± 1.1	0.634
Rural/Urban (*n*)	64/74	41/37	0.382
BMI, kg/m^2^ (M ± SD)	19.8 ± 3.4	19.1 ± 2.5	0.180
Syncopal events, n (Me [IQR])	2.0 [1.0; 4.0]	N/A	N/A^†^
Presyncope duration, min (Me [IQR])	0.5 [0.3; 1.0]	N/A	N/A^†^
Syncope duration, min (Me [IQR])	1.0 [0.5; 2.0]	N/A	N/A^†^
Postsyncope duration, min (Me [IQR])	30.0 [15.0; 60.0]	N/A	N/A^†^
MCSS, points (M ± SD)	−3.2 ± 1.2	N/A	N/A^†^
SDANN, ms (Me [IQR])	210.0 [144.0; 354.5]	260.5 [178.0; 485.0]	0.882
RMSSD, ms (Me [IQR])	256.5 [153.5; 418.0]	305.5 [197.0; 503.0]	0.744
pNN50, % (Me [IQR])	33.5 [21.5; 42.5]	42.5 [21.0; 47.0]	0.099
**TP, ms**^**2**^ **(Me [IQR])**	**7723.0 [4368.0; 11748.0]**	**5471.0 [3853.0; 7067.5]**	**0.012**
VLF, ms^2^ (Me [IQR])	3281.0 [964.0; 4980.0]	4061.0 [2600.0; 5289.0]	0.277
LF, ms^2^ (Me [IQR])	2099.0 [1178.0; 3813.0]	2056.0 [1580.0; 2929.0]	0.114
HF, ms^2^ (Me [IQR])	1286.0 [688.0; 2946.0]	1439.0 [1184.0; 2838.0]	0.799
**LF/HF, units (Me [IQR])**	**1.3 [0.9; 2.1]**	**1.1 [0.9; 1.7]**	**0.039**
**Serum pyridoxine (B6), μg/L (M ± SD)**	**8.9 ± 2.7**	**14.6 ± 4.2**	**<0.001**
Serum folate (B9), μg/L (M ± SD)	4.2 ± 1.3	4.5 ± 1.4	0.252
**Serum cobalamin (B12), ng/L (M ± SD)**	**315.9 ± 93.5**	**428.5 ± 79.1**	**<0.001**
**Serum 25(OH)D, ng/mL (M ± SD)**	**19.6 ± 4.5**	**31.6 ± 6.2**	**<0.001**

M ± SD, mean ± standard deviation; Me [IQR], median [interquartile range]. Bold values: *p* < 0.05. ^†^These parameters were assessed exclusively in the VVS group; statistical comparison with controls is not applicable. Between-group comparisons performed using Student’s *t*-test (normally distributed variables), Mann–Whitney *U* test (non-normal variables), or *χ*^2^ test (categorical variables).

The FPQ demonstrated excellent data quality, with missing responses below 0.5% and only moderate ceiling effects for the “fruits” and “snacks” scales in both VVS and control groups. Internal consistency was satisfactory across all scales (Cronbach’s *α* ≥ 0.71), with overall α of 0.76 in the VVS group and 0.85 in controls. Discriminant validity was supported by Hedge’s g, indicating meaningful differences between VVS and control participants for vegetables, fruits, meat and fish, and starches, whereas the “dairy” and “snacks” scales showed no significant group differences. Exploratory factor analysis confirmed the construct validity of the FPQ, with all scales demonstrating strong factor loadings (≥0.70), indicating a coherent factor structure. These findings suggest that the FPQ has acceptable psychometric properties for assessing food preferences in adolescents aged 13–17 years with VVS and healthy controls, though further validation in larger and more diverse samples is warranted.

Dietary assessment revealed that adolescents with VVS reported lower preferences for vegetables (3.24 ± 0.63 vs. 3.64 ± 0.40, *p* < 0.001), meat and fish (3.60 ± 0.60 vs. 3.87 ± 0.47, *p* = 0.007), and starches (3.35 ± 0.70 vs. 3.76 ± 0.51, *p* < 0.001), all of which remained significant after FDR correction. Fruit preference was also lower (3.99 ± 0.66 vs. 4.26 ± 0.55, *p* = 0.019), though this did not survive FDR correction. Dairy and snack preferences did not differ significantly between groups (Table 2).

**Table 2:** Comparison of food preferences between VVS and control groups.

FPQ scale	VVS group M ± SD	Control group M ± SD	*p*
Vegetables	**3.24 ± 0.63**	**3.64 ± 0.40**	**<0.001** ^†^
Fruit	**3.99 ± 0.66**	**4.26 ± 0.55**	**0.019**
Meat/Fish	**3.60 ± 0.60**	**3.87 ± 0.47**	**0.007** ^†^
Dairy	3.54 ± 0.44	3.55 ± 0.35	0.957
Snacks	4.02 ± 0.59	3.86 ± 0.62	0.139
Starches	**3.35 ± 0.70**	**3.76 ± 0.51**	**<0.001** ^†^

M ± SD, mean ± standard deviation. Between-group comparisons performed using the Mann–Whitney *U* test. Bold values: *p* < 0.05. ^†^Significant after Benjamini–Hochberg false discovery rate (FDR) correction (*q* < 0.05).

In regression analyses, demographic factors influenced food preferences in VVS. Males showed significantly higher preference for dairy (*p* < 0.001) and meat and fish (*p* = 0.012). Older age was associated with lower starch preference (*p* < 0.001), and urban residence with lower starch preference (*p* = 0.009). Higher BMI correlated with lower preferences for fruit (*p* < 0.001) and vegetables (*p* = 0.008). All aforementioned associations survived FDR correction (Table 3).

**Table 3:** Multiple linear regression analysis of food preferences by sex, age, area of residence, and BMI in VVS group.

Dependent variable	Predictor variable							
	Sex		Age		Area of residence		BMI	
	*β* (SE)	*p*-value	*β* (SE)	*p*-value	*β* (SE)	*p*-value	*β* (SE)	*p*-value
Vegetables	0.06 (0.08)	0.451	−0.12 (0.08)	0.109	−0.03 (0.08)	0.635	**−0.24 (0.09)**	**0.008** ^†^
Fruit	−0.11 (0.09)	0.226	0.08 (0.08)	0.319	**0.20 (0.08)**	**0.018**	**−0.30 (0.09)**	**<0.001** ^†^
Meat/Fish	**0.22 (0.09)**	**0.012** ^†^	−0.02 (0.08)	0.770	0.15 (0.08)	0.076	**−0.18 (0.08)**	**0.031**
Dairy	**0.41 (0.08)**	**<0.001** ^†^	−0.03 (0.08)	0.682	−0.04 (0.08)	0.580	−0.12 (0.08)	0.126
Snacks	0.14 (0.09)	0.122	−0.01 (0.09)	0.916	−0.01 (0.09)	0.905	0.03 (0.09)	0.729
Starches	0.08 (0.08)	0.325	**−0.31 (0.08)**	**<0.001** ^†^	**−0.21 (0.08)**	**0.009** ^†^	−0.15 (0.08)	0.074

*β*, standardized regression coefficient; SE, standard error. Sex: 0 = female, 1 = male. Area: 0 = rural, 1 = urban. BMI: kg/m^2^ (continuous). Bold values: *p* < 0.05. ^†^Significant after Benjamini–Hochberg false discovery rate (FDR) correction (*q* < 0.05).

Associations between food preferences and clinical features of syncope were generally weak. Dairy preference was negatively associated with syncope duration (*p* = 0.008) and MCSS score (*p* = 0.012), though neither survived FDR correction. The only association surviving FDR correction was between starch preference and MCSS (*p* < 0.001). Vegetable, fruit, and meat and fish preferences were not significantly linked to clinical syncope parameters (Table 4).

**Table 4:** Multiple linear regression results for food preferences predictors and some clinical peculiarities of syncope.

Predictor variable	Dependent variable									
	Number of syncopal events		Duration of presyncope		Duration of syncope		Duration of postsyncope		MCSS score	
	*β* (SE)	*p*-value	*β* (SE)	*p*-value	*β* (SE)	*p*-value	*β* (SE)	*p*-value	*β* (SE)	*p*-value
Vegetables	−0.02 (0.12)	0.844	0.10 (0.11)	0.404	−0.22 (0.12)	0.066	0.03 (0.12)	0.785	−0.13 (0.11)	0.258
Fruit	0.16 (0.11)	0.145	−0.20 (0.10)	0.058	0.18 (0.11)	0.087	−0.11 (0.11)	0.341	0.16 (0.11)	0.123
Meat/Fish	0.12 (0.10)	0.232	−0.02 (0.10)	0.853	−0.05 (0.10)	0.645	0.10 (0.10)	0.329	0.13 (0.10)	0.189
Dairy	−0.13 (0.10)	0.213	−0.19 (0.10)	0.061	**−0.27 (0.10)**	**0.008**	−0.00 (0.11)	0.966	**−0.25 (0.10)**	**0.012**
Snacks	0.07 (0.10)	0.454	**−0.21 (0.09)**	**0.021**	0.07 (0.09)	0.480	0.03 (0.10)	0.741	0.05 (0.09)	0.610
Starches	0.00 (0.10)	0.998	0.18 (0.09)	0.054	0.01 (0.10)	0.932	0.16 (0.10)	0.120	**0.35 (0.10)**	**<0.001** ^†^

*β*, standardized regression coefficient; SE, standard error. Bold values: *p* < 0.05. ^†^Significant after Benjamini–Hochberg false discovery rate (FDR) correction (*q* < 0.05).

Regarding vitamin status, vegetable preference was positively associated with cobalamin (*p* = 0.004; FDR-corrected) and folate (*p* = 0.016). Meat and fish preference was strongly associated with higher B6 (*p* < 0.001; FDR-corrected), while dairy preference showed a negative association with B6 (*p* = 0.008; FDR-corrected). Fruit preference was negatively associated with B12 (*p* = 0.008; FDR-corrected). Snack and starch preferences showed no significant associations with serum vitamin levels (Table 5).

**Table 5:** Multiple linear regression results for food preferences predictors and serum concentrations of vitamins B6, B9, B12, and D in patients with VVS.

Predictor variable	Dependent variable							
	Vitamin B6		Vitamin B9		Vitamin B12		Vitamin D	
	*β* (SE)	*p*-value	*β* (SE)	*p*-value	*β* (SE)	*p*-value	*β* (SE)	*p*-value
Vegetables	0.17 (0.15)	0.264	**0.35 (0.14)**	**0.016**	**0.42 (0.14)**	**0.004** ^†^	−0.13 (0.14)	0.385
Fruit	−0.13 (0.16)	0.419	−0.25 (0.14)	0.088	**−0.38 (0.14)**	**0.008** ^†^	0.08 (0.14)	0.568
Meat/Fish	**0.57 (0.14)**	**<0.001** ^†^	0.02 (0.13)	0.877	**0.25 (0.12)**	**0.047**	0.13 (0.13)	0.298
Dairy	**−0.38 (0.14)**	**0.008** ^†^	0.14 (0.13)	0.273	**−0.25 (0.13)**	**0.049**	−0.24 (0.13)	0.065
Snacks	0.04 (0.12)	0.714	0.08 (0.11)	0.454	0.05 (0.11)	0.643	0.12 (0.11)	0.303
Starches	−0.16 (0.14)	0.258	−0.05 (0.13)	0.713	−0.08 (0.13)	0.551	0.19 (0.13)	0.156

*β*, standardized regression coefficient; SE, standard error. Bold values: *p* < 0.05. ^†^Significant after Benjamini–Hochberg false discovery rate (FDR) correction (*q* < 0.05).

Finally, food preferences were differentially associated with HRV parameters. Vegetable preference was positively associated with SDANN (*p* = 0.009) and RMSSD (*p* = 0.026), but negatively associated with HF (*p* = 0.001; FDR-corrected) and positively associated with LF/HF (*p* = 0.010). Meat and fish preference was negatively associated with SDANN (*p* = 0.003; FDR-corrected), RMSSD (*p* = 0.007), and HF (*p* = 0.037), and positively associated with LF/HF (*p* = 0.039). Dairy preference showed negative associations with SDANN (*p* = 0.002; FDR-corrected), RMSSD (*p* = 0.002; FDR-corrected), LF (*p* = 0.033), and LF/HF (*p* = 0.019). Snack preference showed modest positive associations with TP (*p* = 0.014), VLF (*p* = 0.025), and LF/HF (*p* = 0.036), though these did not survive FDR correction. Fruit and starch preferences were not significantly related to HRV (Table 6).

**Table 6:** Multiple linear regression analysis of the associations between food preferences and HRV parameters in VVS group.

Predictor variable	Dependent variable							
	SDANN *β* (SE), *p*-value	RMSSD *β* (SE), *p*-value	pNN50 *β* (SE), *p*-value	TP *β* (SE), *p*-value	VLF *β* (SE), *p*-value	LF *β* (SE), *p*-value	HF *β* (SE), *p*-value	LF/HF *β* (SE), *p*-value
Vegetables	**0.31 (0.12),** **0.009**	**0.26 (0.12),** **0.026**	−0.23 (0.13), 0.065	−0.04 (0.13), 0.715	0.11 (0.13), 0.394	0.01 (0.13), 0.912	**−0.43 (0.12),** **0.001** ^†^	**0.32 (0.12),** **0.010**
Fruit	−0.05 (0.11), 0.64	−0.02 (0.11), 0.863	0.02 (0.11), 0.832	−0.05 (0.12), 0.687	0.04 (0.12), 0.770	−0.14 (0.12), 0.231	0.07 (0.11), 0.542	0.05 (0.11), 0.685
Meat/Fish	**−0.29 (0.10),** **0.003** ^†^	**−0.27 (0.10),** **0.007**	−0.01 (0.10), 0.955	−0.07 (0.11), 0.552	−0.10 (0.11), 0.357	0.15 (0.11), 0.179	**−0.21 (0.10),** **0.037**	**0.21 (0.10),** **0.039**
Dairy	**−0.32 (0.10),** **0.002** ^†^	**−0.32 (0.10),** **0.002** ^†^	−0.19 (0.11), 0.078	−0.06 (0.11), 0.584	−0.08 (0.11), 0.494	**−0.23 (0.11),** **0.033**	0.03 (0.10), 0.802	**−0.25 (0.10),** **0.019**
Snacks	0.16 (0.09), 0.078	0.11 (0.09), 0.215	0.03 (0.10), 0.734	**0.25 (0.10),** **0.014**	**0.23 (0.10),** **0.025**	0.19 (0.10), 0.060	0.18 (0.09), 0.054	**0.20 (0.09),** **0.036**
Starches	−0.01 (0.10), 0.977	−0.02 (0.10), 0.854	0.08 (0.10), 0.430	0.07 (0.11), 0.545	−0.04 (0.11), 0.743	−0.09 (0.11), 0.392	0.10 (0.10), 0.296	−0.10 (0.10), 0.318

*β*, standardized regression coefficient; SE, standard error. Bold values: *p* < 0.05. ^†^Significant after Benjamini–Hochberg false discovery rate (FDR) correction (*q* < 0.05).

## Discussion

This study provides an integrative analysis of food preferences, micronutrient status, and autonomic function in adolescents with VVS, highlighting factors beyond classical hemodynamic mechanisms. While VVS is traditionally recognized as a disorder of reflex-mediated cerebral hypoperfusion, our findings suggest that nutritional and behavioral factors may also influence autonomic regulation and syncope susceptibility.

In our study, adolescents with VVS exhibited significant HRV alterations compared with controls, particularly in TP and sympathovagal balance (LF/HF index), aligning with previous pediatric findings (15–17). Prior research reports reductions in time-domain indices such as SDANN and RMSSD, reflecting diminished autonomic flexibility and vagal regulation (1, 2, 18), although in our cohort these differences did not reach statistical significance. Frequency-domain analyses have shown elevated LF, decreased HF, and higher LF/HF ratios, indicating relative sympathetic predominance at baseline (16, 17, 19). These observations are consistent with the pathophysiology of VVS, in which exaggerated autonomic reflexes to orthostatic or sensory stimuli provoke paradoxical bradycardia and vasodilation (20, 21), highlighting the potential utility of baseline autonomic profiling to identify adolescents at higher risk and guide individualized interventions.

Emerging evidence indicates that micronutrient status, particularly vitamin D and B vitamins, may influence autonomic control and vascular responsiveness, key factors in syncope pathophysiology. In our cohort, adolescents with VVS had lower 25(OH)D, serum pyridoxine (B6), and cobalamin (B12) compared with controls, while folate levels were similar. Multiple studies confirm a high prevalence of vitamin D deficiency in pediatric VVS; children with VVS exhibit lower 25(OH)D levels than healthy peers (3), and large epidemiological analyses show higher rates of deficiency in VVS patients (22). Furthermore, VVS children with vitamin D deficiency demonstrate reduced parasympathetic HRV measures, such as lower RMSSD, linking hypovitaminosis D to impaired cardiac autonomic function (1) and potentially increasing syncope susceptibility.

Serum B vitamin profiles have also been linked to syncope characteristics in children with VVS, with lower pyridoxine and cobalamin levels compared with healthy peers (6, 23–25). Although folate differences were not consistently significant, associations with clinical severity suggest that B vitamin insufficiency may contribute to dysautonomia, with elevated homocysteine further implicating disrupted B vitamin metabolism in autonomic dysfunction (23, 26, 27). Importantly, recent interventional studies demonstrate that correcting these micronutrient insufficiencies can improve both clinical and autonomic outcomes. A 2025 trial showed that combined supplementation with vitamins B6, B9 (folate), B12, and D over 3 months significantly reduced syncopal episodes and improved autonomic function scores (7), underscoring the potential therapeutic role of targeted nutritional interventions in mitigating dysautonomia in pediatric VVS.

The observed differences in food preferences between adolescents with VVS and controls are novel in the field and align with recent work exploring eating behaviour patterns in this population. The FPQ demonstrated robust reliability and validity in adolescents with VVS, with minimal missing data, satisfactory internal consistency (*α* ≥ 0.71), and strong construct and discriminant validity. These results support its use for assessing dietary preferences and exploring potential links between habitual food preferences and autonomic regulation. Lower preferences for vegetables, fruits, meat and fish, and starches may reinforce suboptimal micronutrient status, contributing to the biochemical deficiencies observed. Behavioral patterns, including lower food enjoyment and higher satiety responsiveness, have been linked to lower neurotropic vitamin levels and autonomic imbalance in adolescents with VVS (10), suggesting an interaction between eating behaviors and physiological manifestations of VVS.

Regression analyses further revealed that demographic factors were significantly associated with distinct food preference patterns. Male participants preferred meat and fish, and dairy products, consistent with evidence that food preferences vary by sex and age, reflecting biological taste differences and social influences (28–30). Older children showed lower preference for starchy foods, indicating age-related shifts in taste and health awareness (31, 32). Urban residence correlated with higher fruit preferences and lower starch preference, consistent with environmental and sociodemographic influences on dietary patterns (33, 34). Higher BMI was associated with lower preferences for vegetables, fruits, and meat and fish, paralleling research linking poor dietary quality with greater adiposity in pediatric populations (31, 35, 36). Collectively, these findings suggest that sex, age, residence, and BMI shape dietary choices, which may influence nutrient status and autonomic regulation (37, 38).

Overall, food preferences showed limited associations with syncope severity. Most dietary patterns were unrelated to the frequency or duration of syncope or presyncope episodes, consistent with VVS being primarily driven by autonomic reflex mechanisms. However, higher dairy food preference was modestly associated with shorter syncope duration and lower MCSS scores, suggesting a potential protective effect, possibly related to calcium or vitamin D intake (1, 3). Snack and starch preferences showed weak associations with presyncope duration and MCSS, whereas fruit, vegetable, and meat and fish preferences were not significantly related to clinical parameters, highlighting that dietary habits likely act as modifying rather than primary determinants of VVS expression.

In adolescents with VVS, food preferences were selectively associated with serum vitamin levels. Higher vegetable preference was associated with higher folate and cobalamin, while meat and fish preferences were strongly associated with higher B6 and B12, reflecting the contribution of these foods to micronutrient status (39). In contrast, higher fruit preference was inversely associated with B12, while higher dairy preference was inversely associated with B6 and B12, likely reflecting dietary substitution, where these foods displace meat and fish, the principal sources of these vitamins. Snack and starch preferences showed no significant associations with vitamin levels.

Although direct research on diet and HRV in pediatric syncope is limited, evidence from broader nutritional studies suggests that higher intake of key micronutrients is associated with improved HRV, likely through enhanced vagal activity, reduced inflammation, and modulation of cardiac autonomic function (40–42). While not specific to VVS, these findings provide a framework for understanding how adequate status of vitamins B6, B12, folate, and D may influence autonomic regulation and potentially modulate syncope susceptibility.

Our analysis of food preference patterns showed that higher vegetable preference was associated with increased SDANN and RMSSD, suggesting enhanced autonomic regulation, although the negative association with HF indicates a complex effect on parasympathetic activity. Meat and fish preferences were negatively associated with SDANN, and RMSSD, and positively associated with LF/HF, and dairy preference correlated negatively with multiple HRV indices, possibly reflecting dietary displacement or nutrient bioavailability effects. Snack preferences showed modest positive associations with TP, VLF, and LF/HF, whereas fruit and starch preferences had no consistent impact. These results indicate that food preference patterns may reflect underlying dietary tendencies that modulate baseline autonomic tone and sympathovagal balance in pediatric VVS, complementing the pathophysiological profile of exaggerated autonomic reflexes, and suggest that nutritional factors could be considered when identifying high-risk adolescents and planning individualized interventions.

These findings have several clinical implications. First, integrating dietary assessment and micronutrient evaluation into routine care for adolescents with VVS could help identify modifiable risk factors that contribute to autonomic dysfunction, given the high prevalence of suboptimal diet quality and micronutrient deficiencies in this population. Second, interventions targeting nutrient deficiencies—through dietary counseling and, when appropriate, supplementation—may serve as adjunctive strategies to improve autonomic regulation and reduce symptom burden, complementing established management approaches such as increased hydration, salt supplementation, and physical counterpressure maneuvers (21).

Several limitations should be acknowledged. The cross-sectional design limits causal inference. Importantly, the FPQ measures hedonic responses to food rather than quantitative dietary intake; therefore, the observed associations between food preferences and serum vitamin levels should be interpreted as indirect. Food preference is a necessary but not sufficient determinant of actual consumption, which is additionally influenced by availability, cost, parental feeding practices, and cultural norms. Consequently, these findings reflect potential dietary tendencies rather than confirmed nutrient intake patterns. The sample size was modest and recruited from a single center, potentially limiting generalizability. Additionally, HRV reflects only certain aspects of autonomic function, and other physiological or biochemical measures could provide complementary insights. Finally, while our focus was on key vitamins (B6, B12, folate, D), other micronutrients or dietary factors may also influence autonomic regulation and were not evaluated.

In conclusion, adolescents with VVS demonstrate distinct food preference patterns that are linked to micronutrient insufficiencies and alterations in HRV, reflecting disrupted autonomic function. These findings support a model in which dietary behaviour and nutrient status interact with autonomic regulation, contributing to syncope susceptibility. Incorporating nutritional assessment and targeted interventions into clinical management may help improve autonomic balance and reduce symptom burden in this population. Future longitudinal and interventional studies are warranted to clarify causal relationships and to evaluate the effectiveness of tailored dietary and supplementation strategies in mitigating VVS episodes.

## Data Availability

The raw data supporting the conclusions of this article will be made available by the authors, without undue reservation.
